# MOVPE Growth of GaN via Graphene Layers on GaN/Sapphire Templates

**DOI:** 10.3390/nano12050785

**Published:** 2022-02-25

**Authors:** Kazimieras Badokas, Arūnas Kadys, Dominykas Augulis, Jūras Mickevičius, Ilja Ignatjev, Martynas Skapas, Benjaminas Šebeka, Giedrius Juška, Tadas Malinauskas

**Affiliations:** 1 Institute of Photonics and Nanotechnology, Vilnius University, LT-10257 Vilnius, Lithuania; arunas.kadys@ff.vu.lt (A.K.); dominykas.augulis@ff.stud.vu.lt (D.A.); giedrius.juska@ff.vu.lt (G.J.); tadas.malinauskas@ff.vu.lt (T.M.); 2 Center for Physical Sciences and Technology, LT-10257 Vilnius, Lithuania; ilja.ignatjev@ftmc.lt (I.I.); martynas.skapas@ftmc.lt (M.S.); benjaminas.sebeka@ftmc.lt (B.Š.)

**Keywords:** MOVPE, remote epitaxy, gallium nitride, graphene, lift-off

## Abstract

The remote epitaxy of GaN epilayers on GaN/sapphire templates was studied by using different graphene interlayer types. Monolayer, bilayer, double-stack of monolayer, and triple-stack of monolayer graphenes were transferred onto GaN/sapphire templates using a wet transfer technique. The quality of the graphene interlayers was examined by Raman spectroscopy. The impact of the interlayer type on GaN nucleation was analyzed by scanning electron microscopy. The graphene interface and structural quality of GaN epilayers were studied by transmission electron microscopy and X-ray diffraction, respectively. The influence of the graphene interlayer type is discussed in terms of the differences between remote epitaxy and van der Waals epitaxy. The successful exfoliation of GaN membrane is demonstrated.

## 1. Introduction

In recent years, a novel approach to the growth of III-nitrides has emerged, based on 2D materials, such as graphene, as interlayers between the substrate and the epitaxial layer [1,2,3]. Compared to the usual buffer layers, the graphene interlayer has some advantages owing to the weak van der Waals (vdW) bond at the epilayer/graphene interface; the thermal expansion and lattice mismatch requirements are relaxed [4], resulting in reduced defect density [5,6,7], and the epilayer can be mechanically exfoliated and transferred to any substrate of interest [3,8,9]. Furthermore, the monolayer graphene does not completely screen the electrostatic potential of the substrate, which enables the epilayer to follow the crystalline template of the substrate [10,11]. The remote epitaxy and graphene-mediated exfoliation have been demonstrated for several material systems, including III-N [8,9,11,12], III-V [10], II-VI [9], transition metal dichalcogenides [13], perovskites [14], and other complex oxides [15].

The critical step in this approach is the graphene layer transfer. Thicker graphene interlayers allow for easier exfoliation of the grown epilayer, since the separation of films tends to occur within graphene layers [3,16] due to weaker bindings between the graphene layers than that between the graphene and the GaN [17,18]. On the other hand, to ensure interaction between the substrate and the epilayer, the graphene interlayer thickness must not exceed two monolayers [1,9,11], which restrains the graphene layer to either monolayer or bilayer thickness. Meanwhile, depending on the transfer method, cracks, wrinkles, residue, and contamination might decrease the quality of the graphene layer [19,20,21], and significantly affect the epilayer growth.

Generally, the transfer of the graphene layer onto the target substrate is conducted in either a wet or a dry manner. The main disadvantages of dry transfer are the appearance of cracks, due to the interaction with hard surfaces, and a relatively high material cost [19]. Cracks in the graphene layer initiate growth through holes followed by lateral overgrowth [22,23]. Therefore, we used the relatively inexpensive wet transfer method to reduce the formation of cracks. Furthermore, to minimize the impact of other graphene defects, we utilized the multiple overlapping stacks of the monolayer graphene. To evaluate the feasibility of such an approach, we compare the growth of GaN on different graphene interlayers: monolayer, bilayer, and multiple stacks of monolayer graphene.

## 2. Materials and Methods

The GaN layers in the studied samples were grown using a low-pressure metalorganic vapor phase epitaxy (MOVPE) in a flip-top close-coupled showerhead 3 × 2” reactor (AIXTRON, Herzogenrath, Germany). Trimethylgallium (TMGa) and ammonia (NH_3_) were used as Ga and N precursors, respectively. The epitaxy process was monitored by an in situ laser reflectometry system operating at 650 nm. Transfer-ready poly(methyl methacrylate)-coated (PMMA-coated) graphene films from two vendors were used to cover the GaN/sapphire templates. Graphene monolayers were obtained from Graphenea Inc. (San Sebastian, Spain), while the bilayer graphene was acquired from ACS Material, LLC (Pasadena, CA, USA). After the transfer, samples were annealed in an oven with a controlled environment.

The structural characterization was performed using X-ray diffraction (XRD, Rigaku SmartLab, Tokyo, Japan). The surface morphology was studied by scanning electron microscopy (SEM, CamScan Apollo 300, Cambridge, UK, now successor Applied Beams, LLC, Beaverton, OR, USA). The surface roughness was evaluated by atomic force microscopy (AFM, Nanonics MultiView 1000, Jerusalem, Israel). The freely distributed WSxM software was used to analyze AFM data [24]. The GaN–graphene interface was investigated using transmission electron microscopy (TEM, FEI Tecnai G2 F20 X-TWIN, Eindhoven, The Netherlands). Raman measurements were performed using a confocal Raman microscope (WITec alpha 300R, Ulm, Germany). The 532 nm laser excitation source, with a power of 1.5 mW, was focused on a 0.8 µm diameter spot on the sample surface. A 600 lines/mm grating was used to record the Raman spectra. The wavenumber axis was calibrated using a polystyrene standard. All the measurements were performed at room temperature.

## 3. Results and Discussion

The initial GaN/sapphire template was prepared by the standard MOVPE growth of the GaN layer on a 2-inch c-plane sapphire substrate (see Ref. [25] for more details). The thickness of the GaN/sapphire template was 2.7 µm; its surface was smooth with the root mean square (RMS) surface roughness value of 0.2 nm, evaluated using AFM.

### 3.1. Graphene Layer Transfer

**Figure 1:**
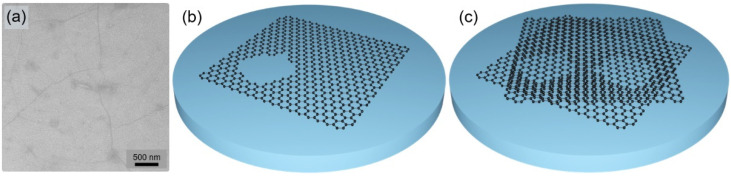
SEM image of monolayer graphene after the transfer procedure (**a**). Illustration of the hole in monolayer graphene (**b**), and of the holes covered by each of two overlapping graphene monolayers (**c**).

Monolayer graphene pieces of size 1.3 cm × 1.3 cm were transferred onto the as-grown GaN/sapphire templates using a wet transfer procedure, described elsewhere [25]. Since graphene transfer might result in the formation of defects [19,20,21], the surface of the transferred graphene layer was checked by SEM. While most of the graphene layer surface was smooth, some wrinkles and possible few-layer zones were observed (Figure 1a). Since direct epitaxy through defects, such as cracks and pinholes, followed by lateral overgrowth [22,23], might significantly aggravate the epilayer exfoliation, to avoid such a growth mode, stacks of two and three graphene monolayers (double-stack and triple-stack, hereinafter) were formed by repeating the complete transfer and cleaning procedures for each monolayer. In this approach, illustrated in Figure 1b,c, overlapping stacks of the graphene monolayer cover the underlying defects, thus reducing the possibility of direct epitaxy. On the other hand, each transfer might introduce additional defects in the overlying layer. 

An alternative to the double-stack graphene might be the bilayer graphene. The wet transfer of bilayer graphene required additional care to prevent roll-up and air becoming trapped. First, the sponge holding the PMMA/graphene in place was soaked by placing small water droplets in the corners. The fully soaked PMMA/graphene/sponge “sandwich” was dipped into deionized water, and the PMMA/graphene was left to float freely on the water surface for a few hours. To avoid the formation of air bubbles, the deionized water was left to stand still overnight before being dipped. Next, the GaN/sapphire template was placed beneath the floating PMMA/graphene sheet, and it was attached as close as possible to the center of the template. The sample was then left to dry in the air for approximately 30 min. Afterwards, the sample was baked in an oven for 30 min at a temperature of 100 °C under N_2_ atmosphere. The PMMA was removed by dipping the sample into acetone and later into isopropyl alcohol. Both solutions were preheated to 40 °C and gently stirred from time to time. Finally, the samples were annealed for 8 h at 300 °C in a vacuum.

**Figure 2:**
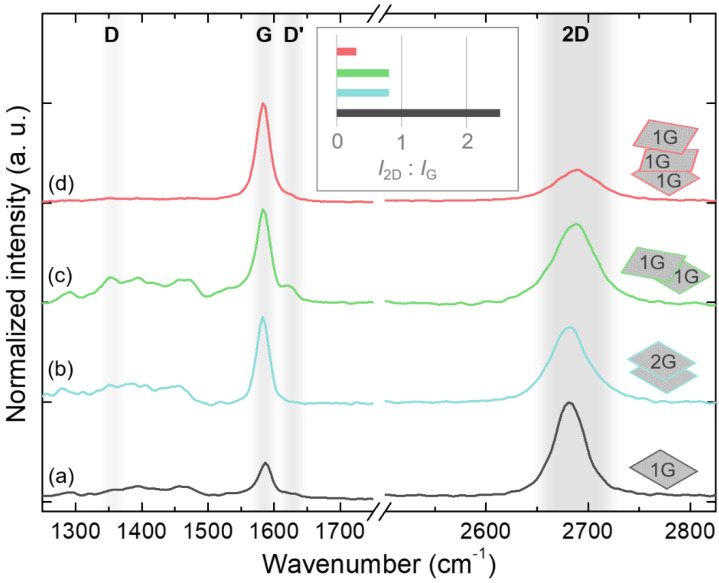
Raman spectra measured after the transfer of monolayer graphene (**a**), bilayer graphene (**b**), double-stack of monolayer graphene (**c**), and triple-stack of monolayer graphene (**d**). The spectra are normalized and shifted vertically for clarity. The approximate positions of the graphene Raman modes: D, D’, G, and 2D are highlighted. Inset illustrates the ratio of 2D and G mode intensities for corresponding layers.

The number and quality of graphene layers on the GaN/sapphire template were verified by Raman spectroscopy. The Raman spectra for all the studied types of graphene interlayer are presented in Figure 2. The graphene Raman fingerprints, G and 2D modes, are prominent in all spectra. The lack of an intense peak at around 1340 cm^−1^ indicated the high quality of the transferred graphene, although the defect-related D mode might be obscured by the second-order peaks of GaN in the broad range of 1250–1500 cm^−1^ [26,27]. The ratio of 2D and G peak intensities (I_2D_/I_G_), the full width at half maximum (FWHM) of a 2D peak, and the position of the 2D peak make it possible to determine the number of graphene layers with a relatively good degree of accuracy [28]. For the transferred monolayer graphene, the ratio I_2D_/I_G_ was 2.5, while the position and FWHM of the 2D peak were 2682 cm^−1^ and 38 cm^−1^, respectively, all consistent with the single graphene layer [28]. For both the double-stack and bilayer graphene, the ratio I_2D_/I_G_ decreased to 0.8, and the 2D peak broadened to 48 cm^−1^, indicating two graphene layers in the film [28]. However, there were some differences related to the main peaks: the 2D peak remained at 2681 cm^−1^, and the G peak slightly shifted to 1583 cm^−1^ in the bilayer graphene. In contrast, the 2D peak shifted to 2687 cm^−1^, and an additional D’ peak emerged on the shoulder of the G peak in the double-stack graphene. The broadening and the shift of the 2D peak are caused by its splitting into different subpeaks, which is explained by the evolution of the electronic bands in graphene with an increasing number of layers [29,30]. The D’ peak could be related to the defects [30], thus indicating the lower quality of the double-stack graphene compared to the bilayer graphene film. Finally, the ratio I_2D_/I_G_ in the triple-stack graphene was reduced to 0.3, which is actually below the expected value of 0.6 [28], and implies layer folding and a formation of zones with a higher number of graphene layers.

### 3.2. Growth of GaN Epilayers

To reveal the impact of the graphene interlayer type on the formation of the initial GaN seeds, the growth of the GaN nucleation layer on the graphene-covered templates was carried out for 5 min at 700 °C without an extra recrystallization process. The selected growth conditions were based on our previous study on the remote epitaxy of GaN [25].

**Figure 3:**
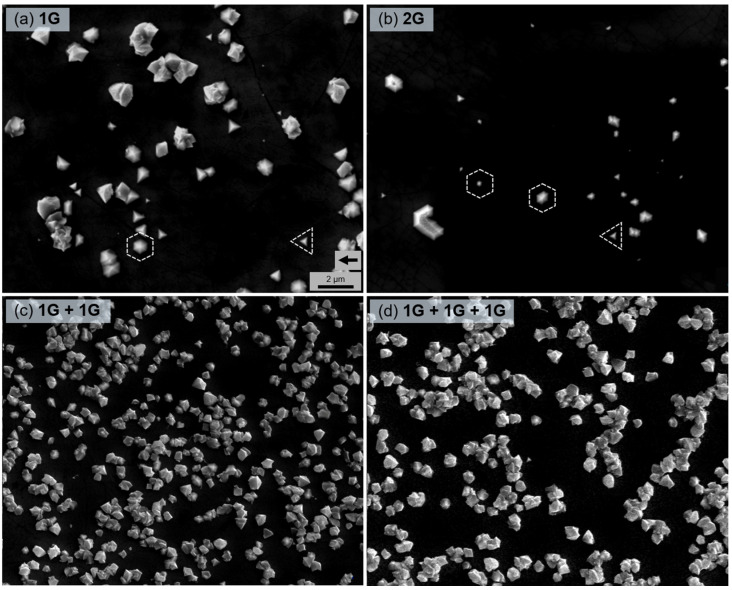
SEM images of the GaN nuclei after the growth of the low-temperature nucleation layer for 5 min on monolayer graphene (**a**), bilayer graphene (**b**), double-stack graphene (**c**), and triple-stack graphene (**d**). Scale bar in (**a**) is the same for all images. The black arrow in (**a**) indicates [$11\overset{¯}{2}0$] direction of an underlying sapphire substrate for all images. White dashed figures illustrate two types of GaN nuclei: hexagonal and triangular.

The initial formation of GaN islands is demonstrated in the SEM images in Figure 3. A certain difference can be noticed immediately: the density of GaN seeds was much higher on the stacked graphene interlayers (Figure 3c,d). It is well known that the lack of dangling bonds and a low surface energy of graphene strongly impede the nucleation process of GaN [31,32]. Therefore, Ga or N adatoms tend to adsorb any defects, where graphene is imperfect and can supply dangling bonds; thus, preferential nucleation sites appear [33,34]. Consequently, the increased density of seeds on stacked layers can be attributed to the graphene layer damage during the transfer, especially considering that several transfers are required for the stacked graphene interlayer.

The epitaxial orientation of the GaN seeds is determined by the electrostatic interaction with the GaN template below the graphene layer [11]. Seeds with aligned crystalline planes were observed on the monolayer and bilayer graphene (Figure 3a,b); however, the GaN islands on stacked graphene seem to be oriented randomly with no specific preferred orientation (Figure 3c,d). A lack of crystalline relationship infers vdW epitaxy, when the substrate field is already screened, instead of remote epitaxy [10,11]. This is expected for the triple-stack graphene interlayer [1,9,11], while the change in the growth mechanism for the double-stack graphene could be related to the formation of zones with a higher number of graphene layers or interface contamination.

**Figure 4:**
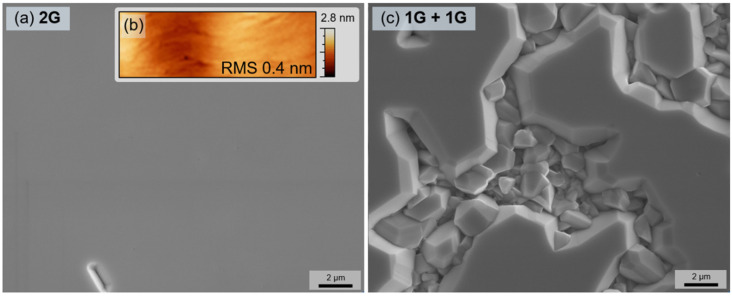
SEM images of the thick GaN epilayers grown on bilayer graphene (**a**) and double-stack of graphene (**c**). To assure that the image in (**a**) is in focus, an arbitrary defect is left visible. The surface morphology of GaN epilayer grown on bilayer graphene is represented by an AFM image within an area of approx. 2 μm × 5 μm (**b**).

To study the difference between bilayer and double-stack graphene interlayers in more detail, the thick GaN layers of 2.5 µm were grown on both interlayer types. The optimized multi-step MOVPE protocol was used to perform nucleation at 700 °C and high-temperature growth at 1075 °C (see Ref. [25] for more details). The growth on the bilayer graphene resulted in a fully coalesced GaN film (surface roughness value of 0.4 nm); however, only a partially coalesced layer was obtained on the double-stack graphene (Figure 4).

**Figure 5:**
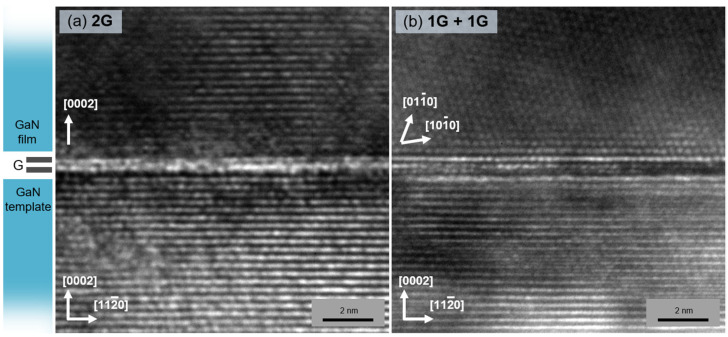
TEM images of GaN film grown on bilayer (**a**) and double-stack (**b**) graphene interlayers. The determined GaN directions are indicated for both the template and the epilayer.

The cross-sectional TEM images (Figure 5) of GaN epilayers revealed the contrasting interfaces in the studied samples. A well-defined two-layer structure of graphene was observed as a bright horizontal strip between the template and the epilayer in the sample with bilayer graphene (Figure 5a). The GaN epilayer on top of the graphene showed coherent atomic steps without significant disordered inclusions. Meanwhile, the double-stack graphene interlayer exhibited an uneven interface (Figure 5b), most likely due to the inner interface contamination or poor adhesion between the two graphene layers. Misoriented GaN crystallites were observed at the interface, confirming the vdW epitaxy growth mechanism, consistent with the SEM images (Figure 4a,c). 

All the presented results indicate the advantages of the single graphene transfer process. Even though multiple transfers of monolayer graphene can be used efficiently to cover the defects and holes in the underlying monolayer, each transfer increases the likelihood of a new defect formation. Eventually, it results in an uneven graphene interlayer with an uncertain thickness, which changes the growth mechanism from remote epitaxy to vdW epitaxy [1,9,11].

**Figure 6:**
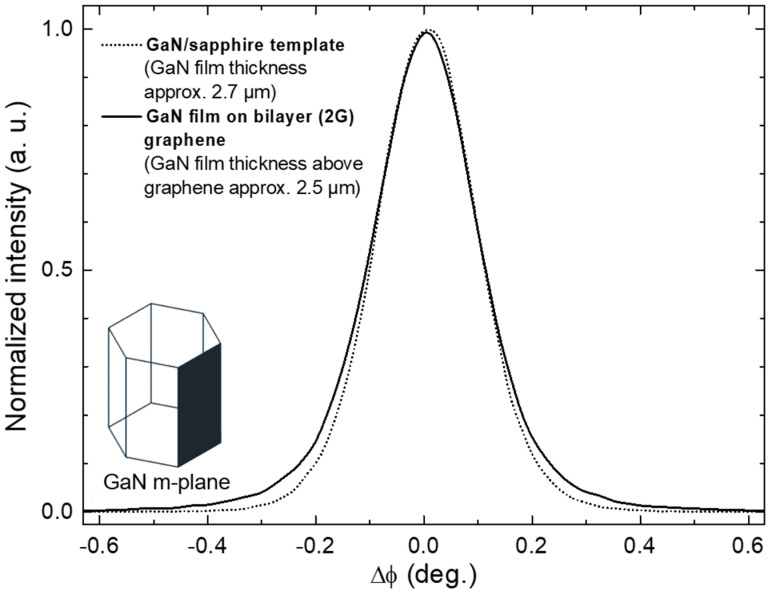
XRD rocking curve of ($1\overset{¯}{1}00$) plane GaN epilayer grown on bilayer graphene (solid line). For comparison, an analogous rocking curve of the GaN/sapphire template is provided (dotted line).

The structural quality of GaN epilayer grown on bilayer graphene was assessed and compared to that of the GaN/sapphire template by using XRD. To minimize the contribution of the underlying GaN template, the structural quality was evaluated using the in-plane geometry of XRD with an incident angle of 0.5 deg. The obtained rocking curves of the ($1\overset{¯}{1}00$) plane are shown in Figure 6 for both the GaN epilayer and the GaN/sapphire template. As evident, the rocking curves are very similar, with the FWHM equal to 767 and 808 arcsec for the template and epilayer, respectively. Since the broadening of the φ-scan of ($1\overset{¯}{1}00$) reflection is affected solely by the edge dislocations, which are dominant in MOVPE-grown GaN epilayers, this indicates the structural quality of the GaN layer grown on bilayer graphene as comparable to the conventional GaN layer deposited on a sapphire substrate using an optimized growth protocol. 

### 3.3. Exfoliation of GaN Epilayer

**Figure 7:**
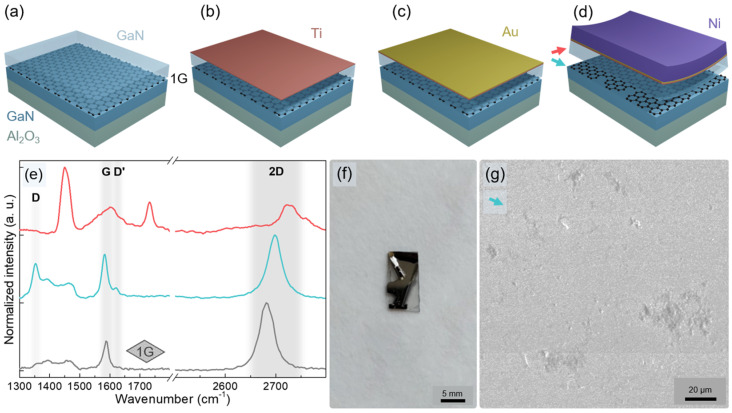
Schematic illustration of metal layer deposition for GaN exfoliation (**a–d**). The thickness of layers is exaggerated. Raman spectra of the template before MOVPE of GaN epilayer (grey line, **e**) and after lift-off of GaN–metal stressor stack (blue line, **e**). Colored arrows in (**d**) indicate the corresponding surfaces where Raman spectra were measured. Raman spectrum of the exfoliated GaN membrane (red line, **e**) (**f**). A stop-motion image of GaN self-exfoliation just after the Ni deposition (**f**). SEM image of graphene interlayer after the GaN epilayer exfoliation (**g**).

For reliable exfoliation of thin GaN films, metal films deposited on the epilayer surface were used to generate the necessary force. The key processes are schematically presented in Figure 7a–d. First, a Ti-based adhesion layer with a thickness of 50 nm was deposited on the GaN surface by e-beam deposition. Next, a 50 nm Au protective layer was deposited using e-beam deposition. To avoid oxidation, both Ti and Au were deposited in a vacuum. Finally, a 5 µm Ni stressor layer was electroplated in a NiSO_4_(H_2_O)_6_ and NiCl_2_ solution. The combination of stresses in electroplated nickel and weak bonding to the interlayer resulted in the start of epilayer exfoliation (Figure 7f). Note that neither low-temperature thermal shock [35] nor additional handling layers [8,36] were required. 

After the exfoliation, the presence of graphene was checked for both the GaN membrane and the remaining template. As revealed by Raman spectroscopy (Figure 7e), graphene survived on the template, although its quality was degraded, which manifested in a strong D peak as well as a visible D’ peak. Meanwhile, the graphene fingerprint peaks were also detected on the GaN membrane. This indicates that the damage to the graphene layer occurs not only during the growth of GaN epilayer [9,37], but also due to the exfoliation. The graphene interlayer was probably ripped during the exfoliation, with some flakes of graphene stuck to the GaN membrane. Furthermore, even though the overall interaction between the GaN/sapphire template and the epilayer is very weak, there might be a direct contact between the epilayer and the template through occasional holes and defects in the graphene layer, which also result in a partially damaged template, as shown in Figure 7g. Nevertheless, the successful exfoliation of the GaN epilayer proves the applicability of the presented approach. The main improvement, however, is due to the wet transfer method, while stacking of graphene monolayers requires further refinement.

## 4. Conclusions

GaN epilayers were grown on GaN/sapphire templates via graphene interlayers. The approach of multiple overlapping stacks of monolayer graphene was utilized to minimize the possibility of growth through holes. To evaluate the impact of interlayers on further growth, different interlayer types were studied: monolayer, bilayer, double-stack of monolayer, and triple-stack of monolayer graphene. Raman measurements revealed a small quality difference between the bilayer and the double-stack graphene, and indicated a higher than expected number of layers in triple-stack graphene. Studies of initial GaN nucleation split the interlayer influence into two groups: low density of aligned islands was observed on monolayer and bilayer graphene, and the high density of randomly oriented islands was observed on stacked graphene interlayers. The different nucleation mechanisms indicated remote and vdW epitaxy, respectively. Further growth of the thick GaN epilayer on the bilayer and double-stack graphene resulted in a high-quality and only partially coalesced GaN epilayer, respectively. Thus, the main focus should be on the graphene transfer: while multiple transfers allow the covering of holes and major defects, the single transfer process leads to a higher quality of resulting epilayer. The weak interaction between the epilayer and the underlying template allowed successful exfoliation of GaN membrane.

## Data Availability

The data that support the findings of this study are available from the corresponding author upon reasonable request.
